# Effects of brown seaweed extract, silicon, and selenium on fruit quality and yield of tomato under different substrates

**DOI:** 10.1371/journal.pone.0277923

**Published:** 2022-12-08

**Authors:** Peyman Jalali, Hamid Reza Roosta, Mohsen Khodadadi, Ali Mohammadi Torkashvand, Marzieh Ghanbari Jahromi

**Affiliations:** 1 Department of Horticulture and Agronomy, Science and Research Branch, Islamic Azad University, Tehran, Iran; 2 Department of Horticultural Sciences, Faculty of Agriculture and Natural Resources, Arak University, Arak, Iran; 3 Vegetable Research Institute, Horticultural Science Research Institute, Research Organizations, Agricultural Research, Education and Extension Organization, Karaj, Iran; University of Agriculture Faisalabad, PAKISTAN

## Abstract

Tomatoes (*Lycopersicun esculentum* L.) are an important group of vegetable crops that have high economical and nutritional value. The use of fertilizers and appropriate substrates is one of the important strategies that can assist in increasing the yield and quality of fruits. The present study aimed to investigate the effects of exogenous seaweed extract (*Nizamuddinia zanardinii*), silicon (Na_2_SiO_3_), and selenium (Na_2_SeO_3_) on quality attributes and fruit yield (FY) of tomato under palm peat + perlite and coco peat + perlite substrates. Seaweed extract significantly improved several of the fruit quality attributes such as total carbohydrate content, total soluble solids (TSS), and pH as well as the FY. The results showed that silicon (Si) (75 mg) was the best foliar spray treatment to enhance the fruit firmness (30.46 N), fruit volume (196.8 cm^3^), and FY (3320.5 g). The highest amount of plant yield (3429.33 g) was obtained by the interaction effects of silicon (75 mg L^-1^) under the effect of palm peat. The use of selenium (Se) led to improvements in flavor index (TSS/TA). Also, the application of palm peat + perlite substrate caused an increase in vitamin C (16.62 mg/100g FW), compared to other substrates (14.27 mg/100g FW). The present study suggested that foliar spray with seaweed extract and Si had beneficial effects on the quality and FY of tomatoes. Also, the palm peat substrate can be used as a good alternative to the coco peat substrate in the hydroponic system.

## 1. Introduction

Tomato (*Lycopersicun esculentum* L.) is one of the important vegetable crops and a popular product in many parts of the world. Tomato, as a major source of lycopene, is rich in potassium and vitamins and is an important component in the human diet [1]. To increase the quality of different fruit, so far, there have been strategies for the application of non-essential elements and fertilizers that contain amino acids, the increase or decrease in some of the macro-elements, accurate control of irradiance, and monitoring temperature conditions, as well as new molecular and genetic engineering methods [2]. The application of silicon (Si) and selenium (Se) in agriculture have been studied for decades. Application of Si and Se with low concentrations reportedly increased the activity of antioxidant enzymes, enhanced plant growth as well as plant yield, and improved tolerance to abiotic stresses [3]. A previous study showed that the application of Se and Si in the forms of sodium selenate (Na_2_SeO_4_) and sodium silicate through either foliar spraying or soil drenching in the root area enhanced the shelf life and quality of strawberries [4] and cucumber [5]. Zhu et al. [6, 7] showed that spraying Se (1 mg L^-1^ sodium selenite) maintained tomato fruit quality by decreasing the production of reactive oxygen species (ROS) and increasing the expression of proteins involved in amino acid metabolism, carbohydrate metabolism, and secondary metabolism. Previous research showed that Se fertilizer improved yield and quality in various plants such as curly endive [8] and tomato [9] in response to adverse environmental conditions by a boost of antioxidant systems and photosynthetic rates. The use of Se in hydroponic solution significantly affected the appearance and quality of cherry tomatoes and led to enhancements in vitamin C, lycopene, and soluble sugar contents of the fruits [10]. Also, Liu et al., [11] revealed that selenium fertilizer positively influenced the mineral composition and flavor quality of tomato plants. So far, a range of experiments has indicated that foliar spraying with Si assisted in the growth and development of both monocotyledons and dicotyledons plants by improving the activity of antioxidants, exudation of phenolic compound, and organic acid anion, while also contributing to the absorption of minerals, accumulation of compatible solutes, maintenance of the water status, and the control of plant growth regulators [3, 12, 13]. Zhang et al., [14] reported that exogenous silicon spraying plays a role in the mitigation of drought stress in tomato seedlings by modulating some genes involved in photosynthesis and regulation of the photochemical process, thereby promoting photosynthesis. Also, Khan et al. [15] showed that Si increased the expression of genes related to antioxidant enzyme activity (peroxidase, ascorbate peroxidase, superoxide dismutase, and catalase). Sayed et al. [3] stated that the characteristics of fruit quality among tomatoes, such as total soluble solids, ascorbic acid, fruit length, and firmness, were improved when plants were sprayed with foliar applications of Si. These findings show that the application of Se and Si may reinforce protective properties in plants against biotic and abiotic stresses, thereby improving the yield and quality of fruits.

As a biostimulant and an extensive, inexpensive resource, seaweeds occur along coastal agricultural areas. Seaweed extract is obtained from *Sargassum liebmannii*, *Ulva lactuca*, *Padina gymnospora*, and *Caulerpa sertularioides*. It is biochemically comprised of macronutrient and micronutrients, amino acids, growth hormones, glycine betaines, protein hydrolysates, vitamins, and algal extract [16].

Previous studies have shown that the application of seaweed extract was beneficial to crop growth and development. The researchers reported that spraying seaweed extract had positive impacts on the accumulation of mineral elements and photosynthesis pigments [17]. Foliar spraying seaweed extract on onion reportedly increased the absorption of N, P, and K by 116, 113, and 93% in comparison with the control plants [17]. The application of seaweed extract effectively improved stress tolerance and yield in various crops, such as tomato [18] and sugarcane [19].

The application of suitable growing substrates in hydroponic systems is essential for achieving high levels of production in crops under greenhouse conditions. The selection of suitable inorganic or organic substrates for hydroponic systems is one of the important points in successful cultivations due to economic and technical effects. The date palm (*Phoenix dactylifera L*) is one of the strategic crops in southern Iran. The waste of date palm includes its branches, stem barks, fronds, and leaves. While it has a higher value of water holding capacity than coco peat substrate, it is most commonly produced by pruning date palm trees each year [20]. According to previous reports, the performance of palm-date wastes as substrate was similar to, or even better than, peat, perlite, and coco peat substrates [21, 22].

Considering the advantages of using silicon (Na_2_SiO_3_), selenium (Na_2_SeO_3_), and seaweed extract that help increase the performance and quality of various products, we monitored tomato plants as they grew in two different substrates after foliar spraying them with silicon, selenium, and seaweed extract under controlled conditions. The objectives of this study were to investigate: (i) the effects of foliar spraying silicon, selenium, and seaweed extract at different concentrations on the yield and quality of tomato; (ii) the effects of two different combinations of substrates (i.e. palm peat + perlite and coco peat + perlite substrates) on the yield and quality of tomatoes.

## 2. Materials and methods

### 2.1. Plant material and treatments

The seeds of tomatoes were sown in polyvinyl chloride tubes (6 cm in diameter and 10 cm in length) in March 2020. After a month, the seedlings (4-leaf stage) were transferred to 8-liter pots. The experiment was a factorial type and was based on a completely randomized design with five replications. The factors included substrates (i.e. coco peat + perlite and waste of date palm + perlite) as well as seven treatments of foliar spraying, i.e. the control (normal conditions), two levels of Na_2_SiO_3_ (25 and 75 mg L^-1^), two levels of Na_2_SeO_3_ (4 and 10 mg L^-1^) and two levels of seaweed extract (10 and 20%) with five replications. The pots were filled with coco peat (50%) + perlite (50%) and waste of date palm (50%) + perlite (50%). One tomato plant was cultivated per pot. The plants were maintained in a greenhouse with natural light conditions (13-h photoperiod, with a relative humidity of 28 to 62%, and a photosynthetic photon flux density of 500 μmol.m^–2^.s^–1^ at the canopy level). Plants were fertigated three times a day (200 mL) with Hoagland’s nutrient solution [23] which was delivered at 06:00, 12:00, and 19:00 h. The foliar spraying was carried out with seaweed extract, Na_2_SeO_3,_ and Na_2_SiO_3_, which began to be applied 21 days after transplanting the seedlings and, subsequently, every one week up to 30 days after the transplanting.

### 2.2. Seaweed extracts

Algae *Nizamuddinia zanardinii* were collected in the Gulf of Oman off the coast of Chabahar (25°20’ N, 60°27’ E), Iran. The algae were washed with distilled water and dried for 8 days at room temperature and subsequently ground in a mortar. Then, the aqueous extract was produced by boiling 50 gr of algae powder in 500 mL of distilled water, and the extracts were filtered through a filter paper (Whatman No 1; Whatman, Pittsburgh, PA, USA) before storage [24]. The chemical characteristics of the seaweed are presented in Table 1.

**Table 1:** Chemical composition of seaweed extracts evaluated in tomato production.

Manganese	Copper	Zinc	Iron	Potassium	Phosphorus	Nitrate		
		ppm					pH	color
10	4	6	95	62.3	3.2	12.86	7.2	Brown

### 2.3. Total carbohydrate content

Total carbohydrate contents in tomato fruits were estimated using the Anthrone reagent method [25] with slight modifications. Mature fruits were collected from plants and dried. About 100 mg of dried samples were weighed, followed by adding 10 ml of water into a test tube. The solution was kept in a boiling water bath (90°C) for 50 min. One mL (1.00 mL) of the extract was taken from the test tubes and 4 mL of the Antheron reagent (3%) (200 mg/100 ml of ice-cold 95% sulphuric acid) was added to the extract. The mixtures were kept in a water bath (60°C) for 30 min at 100°C. The samples (2 ml) were taken in cuvettes and the maximum value of absorption was measured at 630 nm using glucose as a standard.

### 2.4. Total soluble solids (TSS), titratable acidity (TA), flavour index, and pH

The juice of flesh tissues (10 gr) was extracted from 8 fruits, using a domestic juice extractor, and then centrifuged at 12,000× rpm for 5 min. The supernatants were dropped onto a handheld digital refractometer. The content of TSS was expressed as % [26].

Total titratable acidity was estimated as described by Khan et al. [26]. Tomato juice was titrated with 0.1 M NaOH to an end point of pH 8.2. TA was expressed as percentages, based on malic acid. The ratio of TSS/TA was calculated as the flavour index [27]. Fruit juice pH was measured with a pH meter (HI-2211; Hanna Instruments, Padova, Italy).

### 2.5. Fruit firmness

The fruit firmness was measured at five spots on each fruit. Four fruits were randomly applied per replicate (5 replications per sampling time) using a handheld penetrometer (FT-327; UC Fruit Firmness Tester, Milano, Italy).

### 2.6. Vitamin C

Vitamin C content was measured as described by Contreras-Calderón et al. [28]. Tomato fruit juices (5 g) were mixed with 15 ml of 2 g/100 g oxalic acid solution and centrifuged at 12,000 rpm for 15 min. Then, 10 mL of the supernatant were titrated to a permanent pink color by 0.1 g/100 g 2, 6-dichlorophenolindophenol titration. Vitamin C content was estimated according to the titration volume of 2,6- dichlorophenolindophenol and expressed as milligrams per 100 g of fresh weight.

### 2.7. Yield

All fruits per plant from each treatment were harvested from the first to the fifth tomato cluster and their fresh weights and fruit volume were determined.

### 2.8. Statistical analysis

In this research, a factorial experiment was conducted based on a completely randomized design with five replications. Analysis of variance (ANOVA) was laid out, and means were compared using the Duncan Multiple Range test by the least significant difference (P = 0.01). The SAS 9.1 software (SAS Institute, Inc., Cary, NC) was used for data analysis.

## 3. Results

### 3.1. Fruit firmness

**Fig 1:**
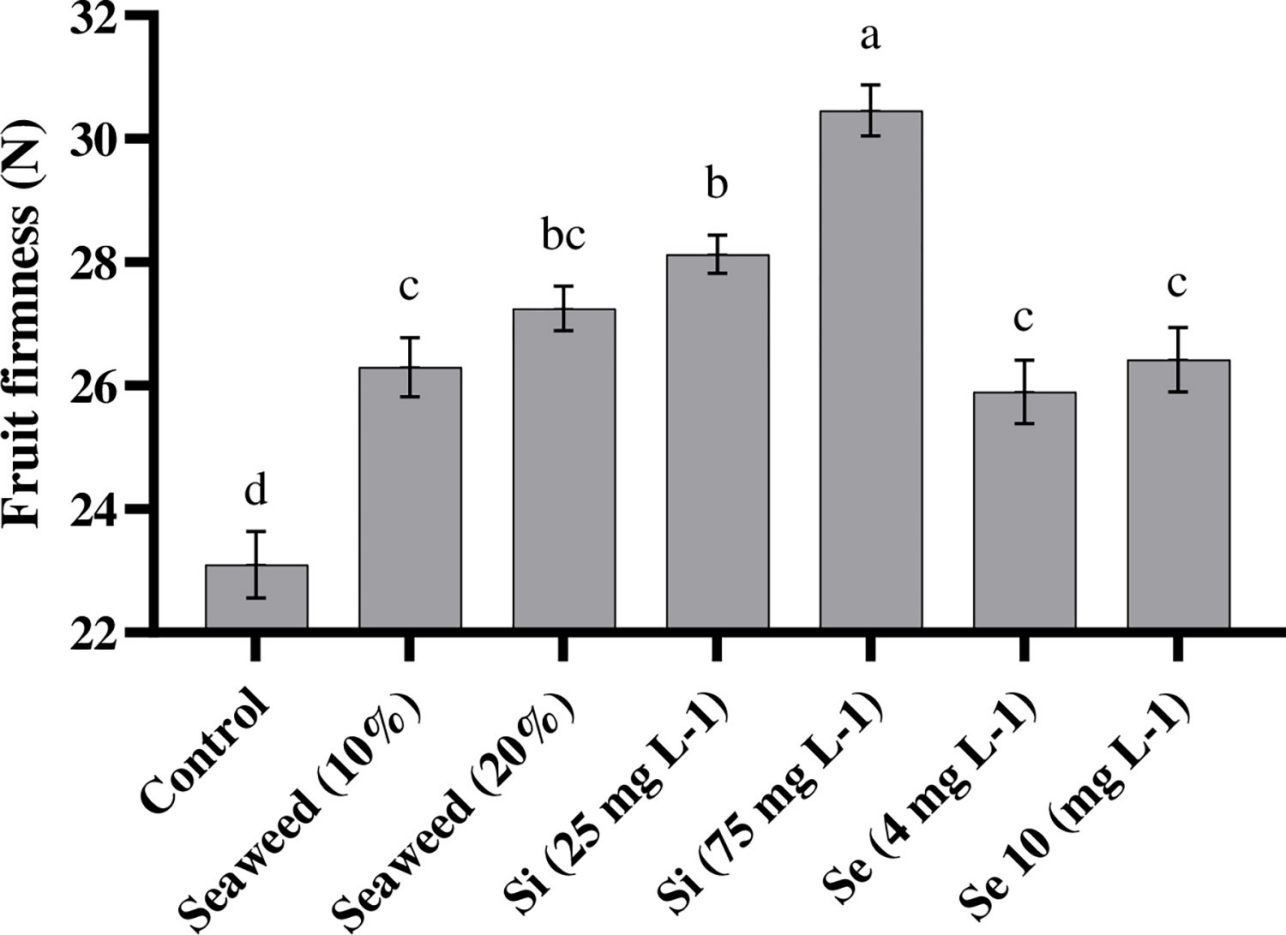
Effects of seaweed extract (10 and 20%), silicon (Si, 25 and 75 mg L^-1^), and selenium (Se, 4 and 10 mg L^-1^) on fruit firmness in tomato plants.

Our results showed that the application of Si, Se, and seaweed extract significantly (P < 0.01) increased the rate of fruit firmness in the tomatoes. The highest value of fruit firmness (30.5 N) was obtained by the interaction effects of Si 75 mg L^-1^ under the effect of palm peat and the lowest value of fruit firmness (22.73 N) was observed in the control treatment (Table 3). Both Si treatments increased fruit firmness and the highest value was obtained in tomato plants that had been exposed to 75 mg L^-1^ Si (Fig 1). In addition, Se and seaweed extract also improved the firmness of the fruit compared to the control treatment. The application of seaweed extract had more beneficial effects on fruit firmness in comparison with the effect of the Se treatment (Table 3).

### 3.2. Total carbohydrate content

**Fig 2:**
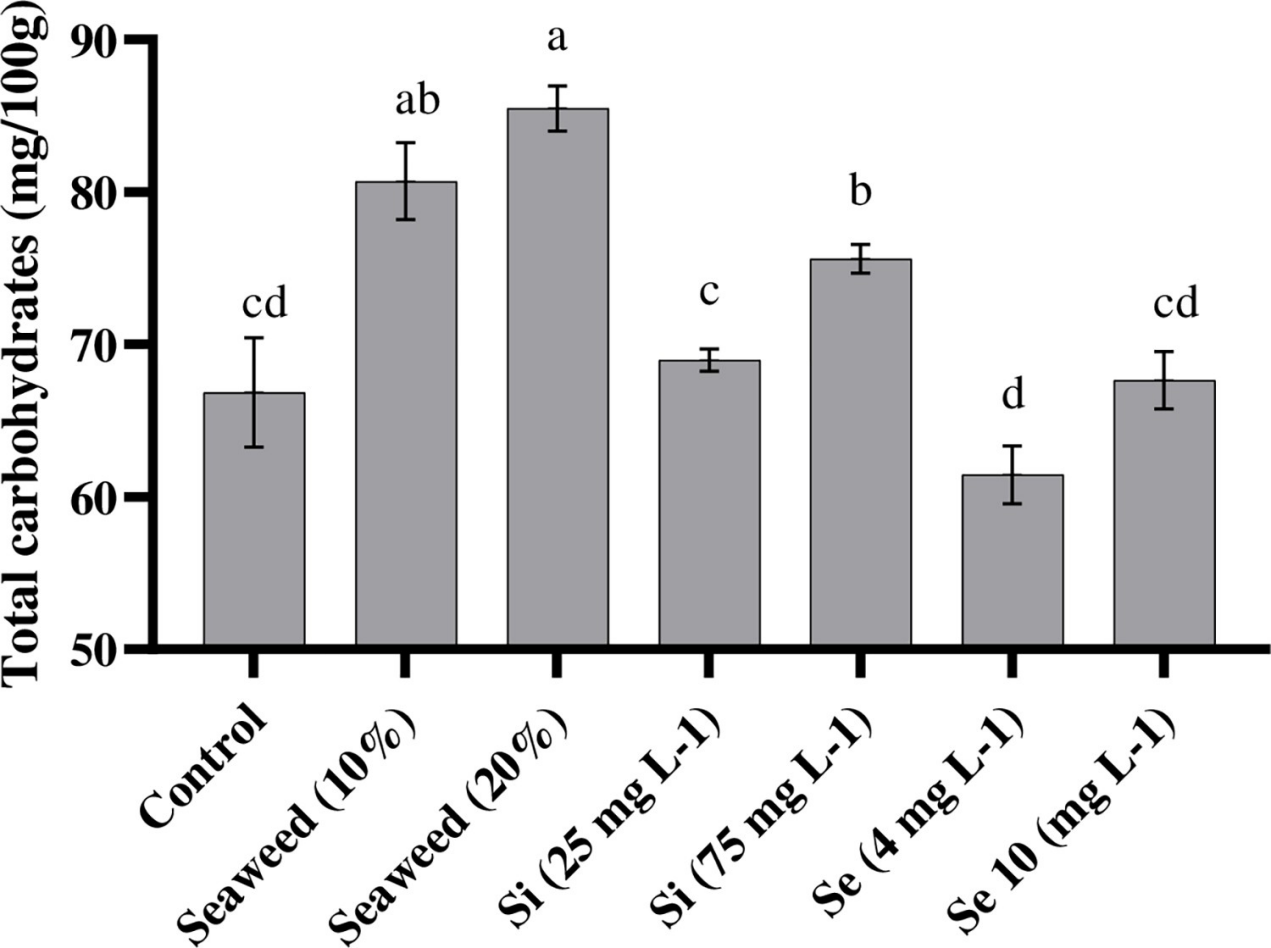
Effects of seaweed extract (10 and 20%), silicon (Si, 25 and 75 mg L^-1^), and selenium (Se, 4 and 10 mg L^-1^) on total carbohydrate content in tomato.

The total carbohydrate content is one of the important traits for the investigation of fruit quality. There was a significant difference among different foliar spray treatments for this measurable feature. Plants that had been treated with seaweed extract had higher (85.49 and 80.71 mg) mean values of this trait, compared with other treatments (Fig 2). As indicated in Fig 2, exogenous 4 mg L^-1^ Se application significantly decreased total carbohydrate content in fruits, which made it significantly lower (61.45 mg/100g) than the control treatment 66.85 mg/100g). Also, 75 mg L^-1^ Si application had useful impacts (75.61 mg/100g) on the total carbohydrate content. According to the results of ANOVA, there were no significant effects of substrate on total carbohydrate content (Table 2).

**Table 2:** Analysis of variance for the measured characters.

S.O.V	df	Means of square								
		FF	TCC	TSS	TA	pH	FI	VC	FV	Y
Substrate (S)	1	0.13 ns	67.1 ns	0.01 ns	0.01 ns	0.001 ns	1.93 *	53.47 **	123.4 ns	42561 ns
Foliar spry(F)	6	30.2 **	435 **	1.64 **	0.01 **	0.27 **	1.21 *	1.38 ns	1036 **	673127 **
F × S	6	0.22 ns	41.1 ns	0.06 *	0.00 ns	0.006 ns	0.46 ns	0.22 ns	47.1 ns	25121 ns
Error	28	1.51	19.4	0.02	0.001	0.009	0.46	0.8	48.24	27358.9
CV (%)	-	8.65	12.6	8.09	9.01	5.99	8.36	9.2	7.9	11.88

FF: fruit firmness, TCC: total carbohydrate content, TSS: total suspended solids, TA: titratable acidity, FI: Flavour index, VC: vitamin C, FV: fruit volume, Y: yield; ns

*, **: non-significant or significant at the 5 and 1 percent levels.

### 3.3. Total soluble solids (TSS)

**Fig 3:**
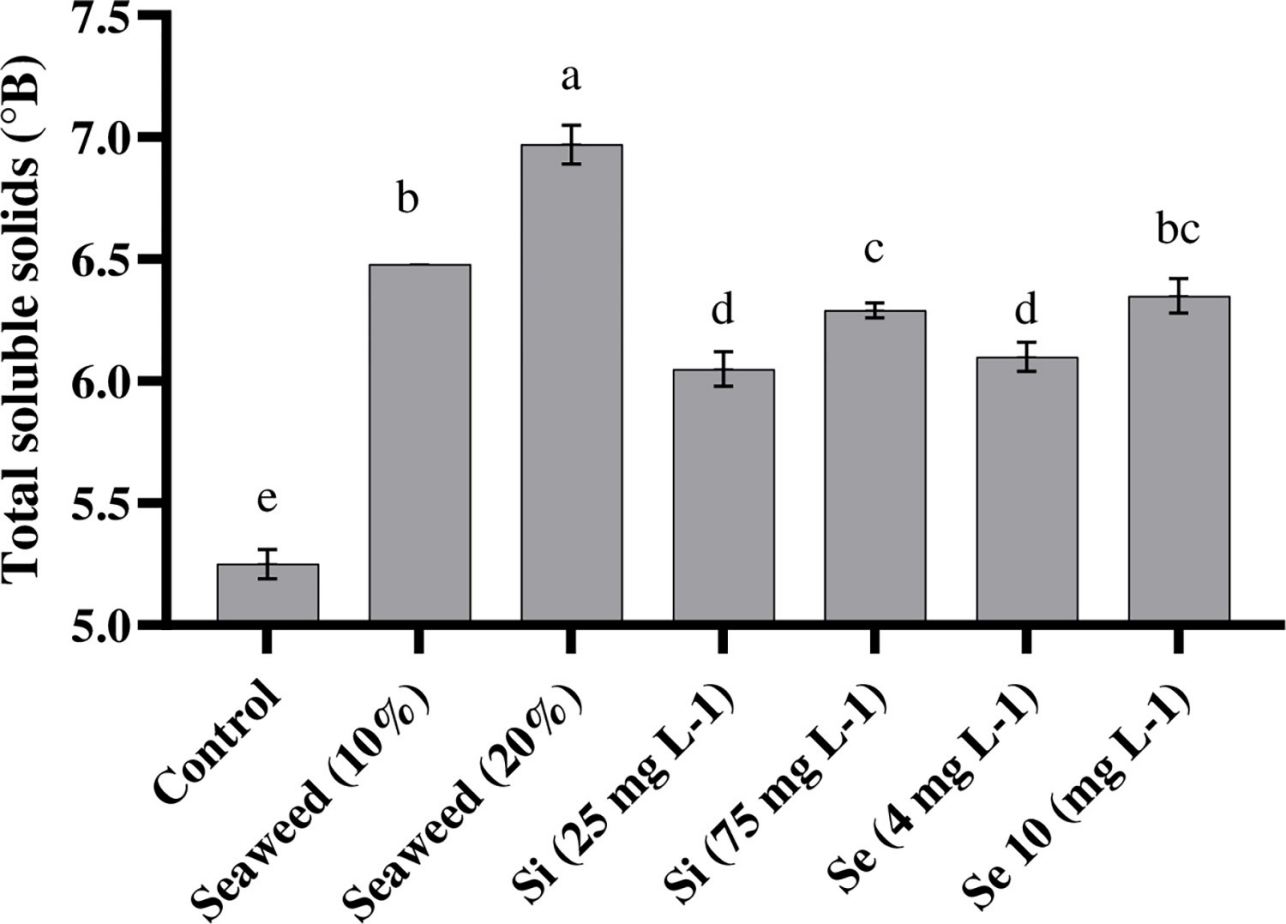
Effects of seaweed extract (10 and 20%), silicon (Si, 25 and 75 mg L^-1^), and selenium (Se, 4 and 10 mg L^-1^) on total soluble solids in tomato plants.

**Fig 4:**
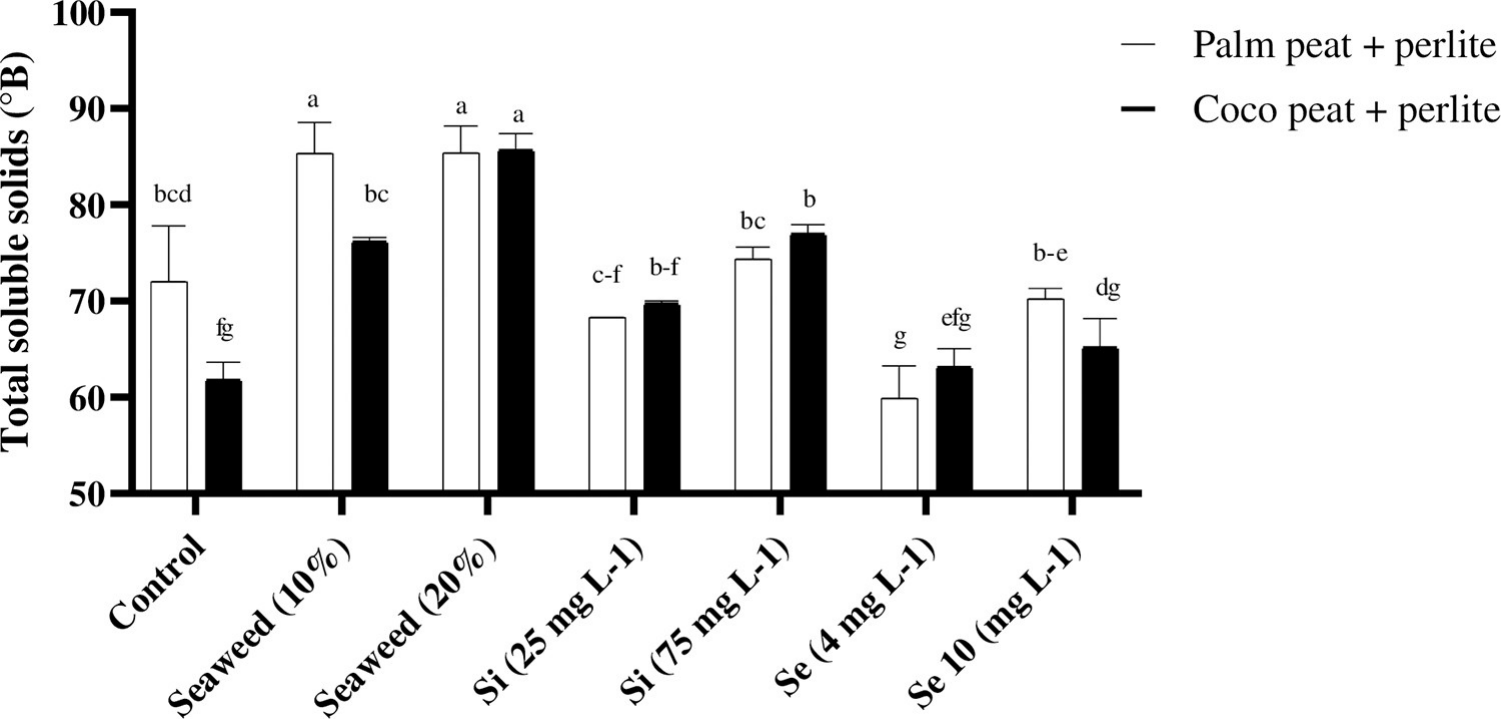
Interaction effects of the foliar spray (seaweed extract (10 and 20%), silicon (Si, 25 and 75 mg L^-1^), and selenium (Se, 4 and 10 mg L^-1^)) with the two substrates on total soluble solids in tomato.

According to the results of the present study, different foliar spray treatments had a significant positive effect on TSS content in comparison with the control (Fig 3). TSS contents were significantly affected by the exposure of plants to different foliar spray treatments. Higher TSS contents were observed in plants that had been exposed to different concentrations of seaweed extract (6.97 and 6.48°Brix) in comparison with the effect of other treatments under both substrates. In this study, the interaction of coco peat substrate and seaweed extract (20%) led to the highest content of TSS, although it had no significant difference with the interactions of date-palm peat substrate and the two levels of seaweed extracts (10 and 20%; Fig 4).

### 3.4. Total titratable acidity and pH

**Fig 5:**
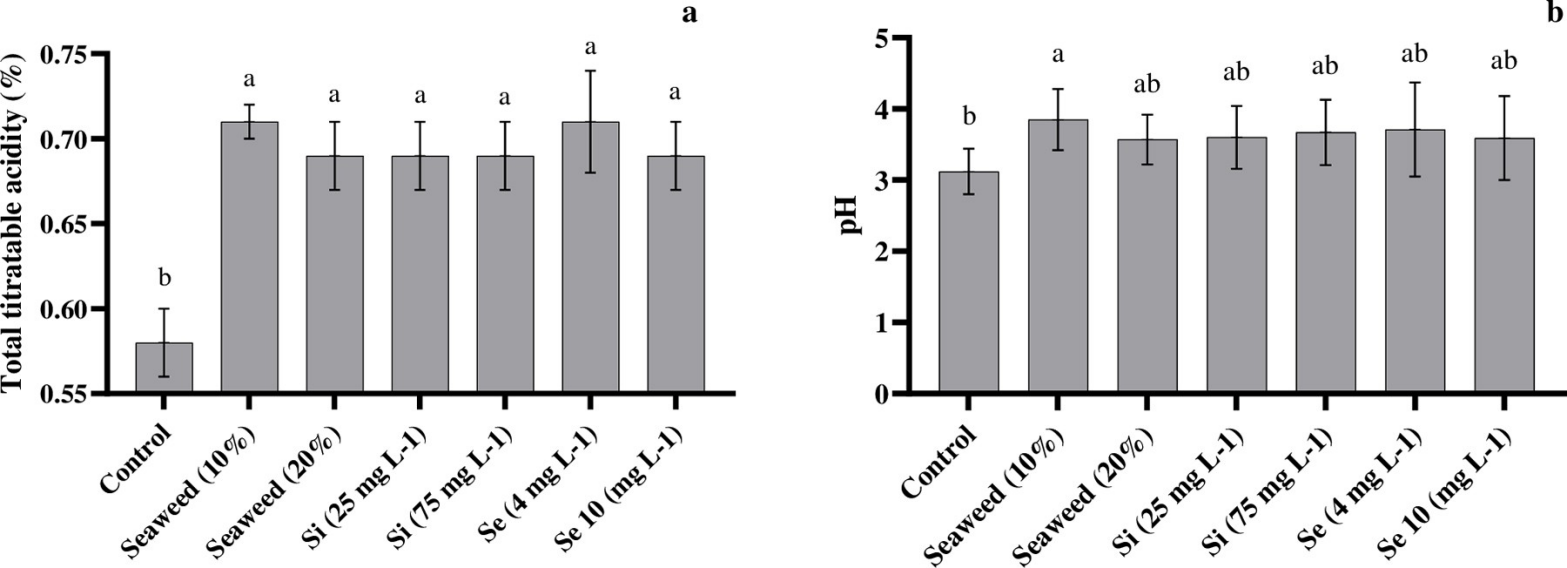
Effects of seaweed extract (10 and 20%), silicon (Si, 25 and 75 mg L^-1^), and selenium (Se, 4 and 10 mg L^-1^) on total titratable acidity (a) and pH of tomato juice (b).

The analysis of variance revealed that the substrate treatments had no significant impact on the amount of titratable acidity (TTA) and pH (Table 2). The amount of TTA and pH of tomato juice were significantly affected by the seaweed extract, Si, and Se, compared with the control, although there was no significant difference among these treatments (Fig 5). The higher mean values in both characteristics were observed in plants that had been exposed to Se (4 mg L^-1^) and seaweed extract (10%), respectively (Fig 5).

### 3.5. Flavour index and Vitamin C

**Fig 6:**
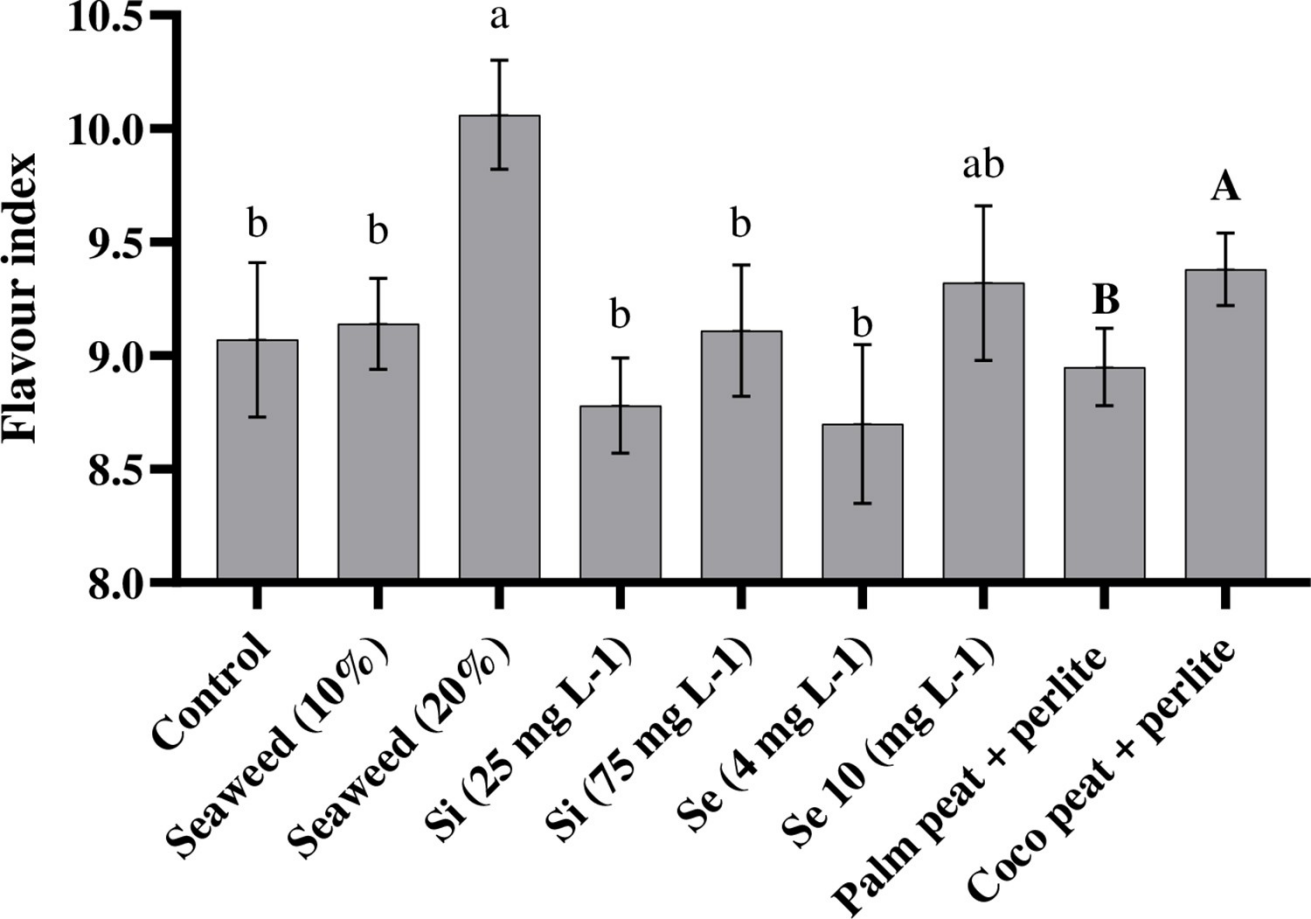
Effects of seaweed extract (10 and 20%), silicon (Si, 25 and 75 mg L^-1^), and selenium (Se, 4 and 10 mg L^-1^) and effects of two different substrates on flavour index (TSS/TA).

**Fig 7:**
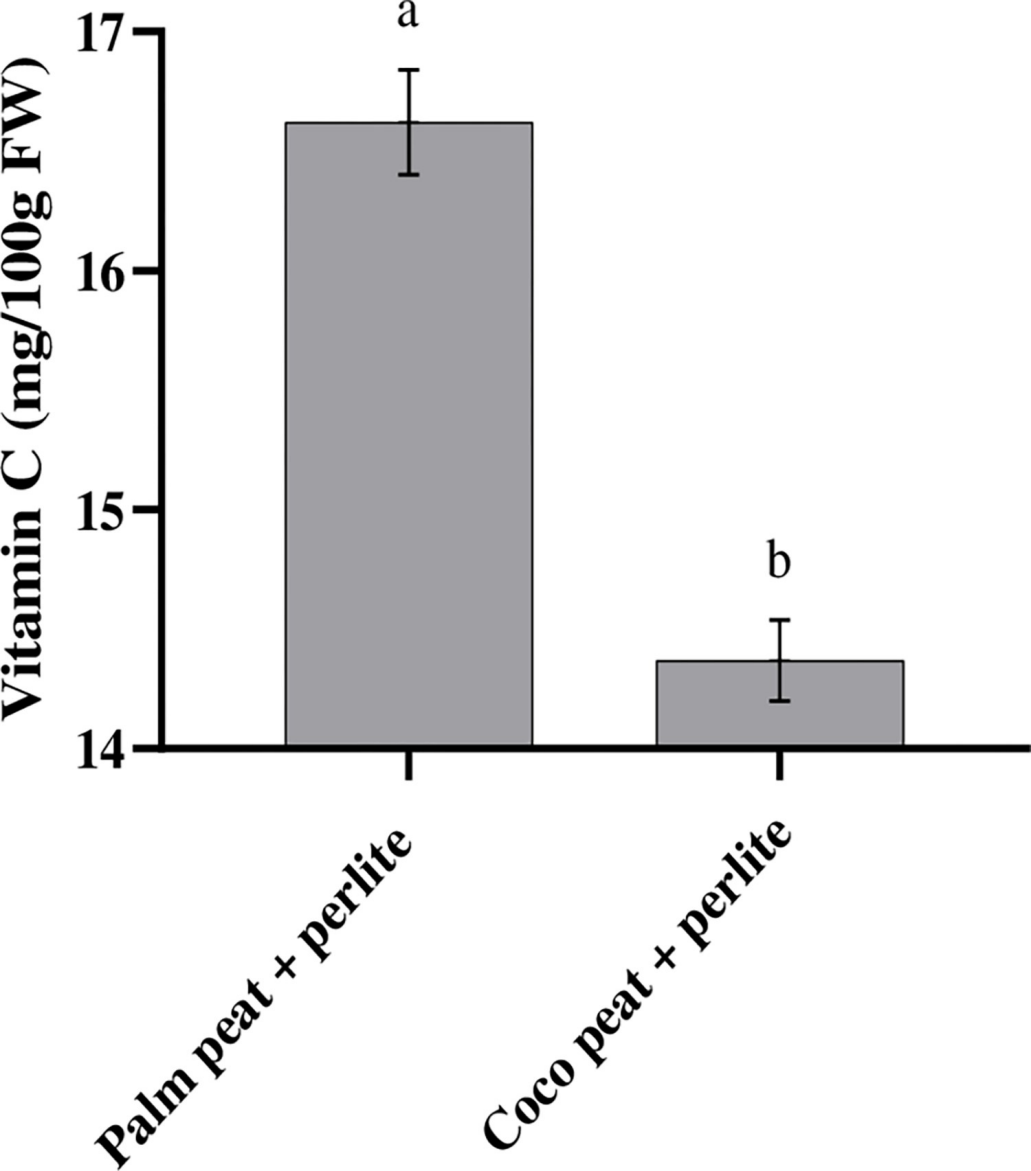
Effect of palm peat + perlite and coco peat + perlite substrates on vitamin C content.

Our results showed that foliar spraying tomatoes with seaweed extract (20%) and Se (10 mg L^-1^) caused higher levels of flavor index than plants foliar sprayed with Si or water (control) (Fig 6). We observed that the application of seaweed extract (20%) led to increases of flavour index from 8.7 to 10. The results revealed that coco peat + perlite substrate had beneficial effects on the flavour index in comparison with palm peat + perlite substrate (Fig 6). As indicated in Table 2, foliar spraying with seaweed extract, Se, or Si, caused no significant difference in the amount of vitamin C. Our results showed that the amount of vitamin C was higher in tomatoes cultivated in the date-palm peat + perlite substrate (17 mg/100g FW) in comparison with coco peat + perlite substrate (14 mg/100g FW; Fig 7).

### 3.6. Fruit volume and yield

**Fig 8:**
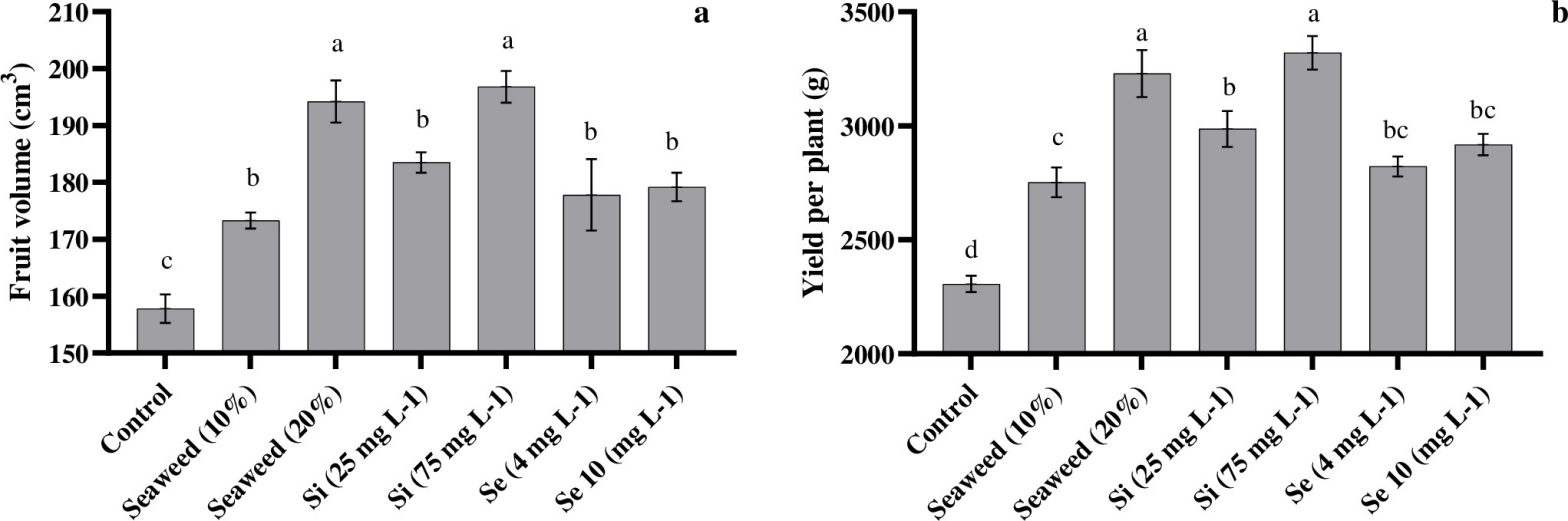
Effects of seaweed extract (10 and 20%), silicon (Si, 25 and 75 mg L^-1^), and selenium (Se, 4 and 10 mg L^-1^) on fruit volume (a) and yield per plant (b).

According to the results, the higher concentrations of seaweed extract and Si increased the FY and fruit volume more, compared to their lower concentrations (Fig 8). The highest value for the plant yield (3429.33 g) was obtained by the interaction effects of Si 75 mg L^-1^ under the effect of palm peat (Table 3). Also, based on the results, all treatments enhanced the FY (Fig 8). However, Si (75 mg L^-1^) and seaweed extract (20%) increased the FY by 70 and 68%, respectively, compared with the control (Fig 8). Our results showed that the application of different substrates caused no significant difference in the FY and fruit volume (Table 2).

**Table 3:** Interaction effects of the foliar spray with the two substrates on different characters in tomato.

Substrate	Foliar spray	FF	TCC	TA	pH	FI	VC	FV	Y
Palm peat	Control	22.73 ± 1.04	72.02 ± 6.69	0.62 ± 0.03	3.12 ± 0.08	8.46 ± 0.28	16.99 ± 1.03	155.67 ± 4.73	2344 ± 11.02
	Seaweed 10%	26.43 ± 1.13	85.34 ± 3.7	0.71 ± 0.02	3.57 ± 0.08	9.15 ± 0.24	16.39 ± 0.74	172.67 ± 0.77	2748.67 ± 146.43
	Seaweed 20%	27.33 ± 0.38	85.41 ± 3.2	0.7 ± 0.03	3.85 ± 0.06	10.27 ± 0.39	16.83 ± 0.61	197 ± 4.06	3326.67 ± 213.05
	Silicon (25 mg L^-1^)	28 ± 0.67	68.33 ± 1.64	0.71 ± 0.02	3.61 ± 0.12	8.54 ± 0.39	17.51 ± 0.64	186.67 ± 2.52	3059.33 ± 111.01
	Silicon (75 mg L^-1^)	30.5 ± 1	74.34 ± 1.46	0.7 ± 0.02	3.67 ± 0.08	9 ± 0.26	15.98 ± 0.47	196.67 ± 5.82	3429.33 ± 136.84
	Selenium (4 mg L^-1^)	25.67 ± 1.02	59.89 ± 3.9	0.73 ± 0.03	3.71 ± 0.04	8.28 ± 0.35	15.98 ± 0.63	166.33 ± 3.67	2771.33 ± 45
	Selenium (10 mg L^-1^)	26.5 ± 0.88	70.23 ± 1.24	0.69 ± 0.03	3.6 ± 0.03	8.97 ± 0.39	16.69 ± 0.57	175.67 ± 4.91	2877.33 ± 55.06
Coco peat	Control	23.47 ± 0.85	61.68 ± 2.28	0.54 ± 0.03	3.13 ± 0.1	9.67 ± 0.48	14.36 ± 0.56	160 ± 3.53	2269 ± 80.75
	Seaweed 10%	26.17 ± 0.51	76.07 ± 0.63	0.71 ± 0.03	3.58 ± 0.04	9.13 ± 0.45	14.51 ± 0.33	174 ± 3.46	2755.33 ± 80.62
	Seaweed 20%	27.17 ± 0.84	85.57 ± 2.1	0.69 ± 0.03	3.71 ± 0.04	9.84 ± 0.4	13.99 ± 0.73	191.33 ± 8.15	3132 ± 114.41
	Silicon (25 mg L^-1^)	28.27 ± 0.43	69.62 ± 0.44	0.67 ± 0.03	3.61 ± 0.04	9.01 ± 0.25	15.32 ± 0.68	180.33 ± 1.02	2913.33 ± 149.06
	Silicon (75 mg L^-1^)	30.42 ± 0.35	76.87 ± 1.25	0.68 ± 0.05	3.72 ± 0.04	9.22 ± 0.7	14.22 ± 0.23	197 ± 4.37	3211.67 ± 41.43
	Selenium (4 mg L^-1^)	26.13 ± 0.8	63 ± 2.4	0.68 ± 0.06	3.66 ± 0.04	9.12 ± 0.69	13.84 ± 0.21	189.33 ± 8.44	2872.33 ± 86.18
	Selenium (10 mg L^-1^)	26.33 ± 1.02	65.07 ± 3.59	0.68 ± 0.04	3.64 ± 0.02	9.68 ± 0.66	14.34 ± 0.41	182.67 ± 0.77	2957.33 ± 98.5

FF: fruit firmness, TCC: total carbohydrate content, TA: titratable acidity, FI: Flavour index, VC: vitamin C, FV: fruit volume, Y: yield.

## 4. Discussion

### 4.1. Fruit firmness

Fruit firmness is one of the main factors in determining the quality of tomato fruits. It is extremely important in overall product acceptance [29]. Extended firmness is favorable for the long-term storage of tomato fruits by consumers. Huang et al. [30] showed that cell size, cell wall structure, turgidity, and membrane properties affected the firmness parameters. Our results showed that foliar spraying with Si substantially enhanced fruit firmness in tomato plants. Similarly, Si led to an increase in the fruit firmness among different plants such as apple, strawberry, avocado, and cherry tomato [31]. It seems that the application of Si probably decreased the rate of tomato softening by slowing down the process of cell-wall degradation while increasing stability in the cell wall structure by causing the binding of Si to the cell wall of pectin molecules [32]. On the other hand, it was reported that the deposition of silicon led to changes in the cell wall and increased the number of non-diffusible anion sites, which adsorb Ca, thereby causing more absorption of Ca by increasing the fruit firmness [33]. Also, we observed that the application of Se on tomato plants improved fruit firmness in comparison with the control. Hernández-Hernández et al. [34] revealed that the application of Se could be a successful strategy for increasing the shelf life of tomato fruits by enhancing fruit firmness. The authors showed that enhancing firmness in tomato fruits, caused by Se, could have resulted from enhancements in the activity of the PAL enzyme and the lignification of the pericarp cell wall. Our results revealed that firmness was positively affected by seaweed extract.

### 4.2. TCC and TSS

The total carbohydrate and TSS contents, as indicators of fruit quality, were recommended by previous studies. The current data showed that foliar spraying with seaweed extract substantially affected the accumulation of TCC and TSS contents in tomato plants. We showed that the increase in seaweed extract concentration positively led to enhancements in the accumulation of TCC and TSS in tomato plants. Similar to our results, it was reported that the application of seaweed extract (*Sargassum johnstonii* or *Ascophyllum nodosum*) led to accumulations of carbohydrates in various plants such as tomatoes [35]. This increase in the TCC of the plants, which had been treated with seaweed extract, may be attributed to the increase in vegetative growth characteristics [36]. Also, the increase in TCC might be attributed to the higher availability of macro and micro elements (K, N, Mg, Ca, Zn, and Ni) and better growth of roots by foliar spraying the seaweed extract [37]. Rasouli et al. [38] reported that the application of seaweed extract increased the photosynthesis rate and chlorophyll index in lettuce plants and, subsequently, caused the accumulation of carbohydrates. On the other hand, our results showed that the application of Si significantly increased the amount of TCC than the control. It was reported that Si had beneficial effects on the photosynthesis of tomato seedlings and the regulation of carbohydrate metabolism in cucumber plants [14].

TSS status of the tomatoes was largely influenced by exposure of plants to different foliar spraying treatments. These data were consistent with previous studies in which seaweed extract (*Ascophyllum nodosum* and *Ecklonia maxima*) enhanced the TSS in *Allium cepa*, *Cucurbita pepo*, and *Vitis vinifera* [39]. Also, Kumari et al. [35] reported that a higher concentration of total soluble sugars in tomatoes was observed in foliar applications of seaweed extract which confirms our results on the useful effect of seaweed extract on TSS content. The TSS content depended on the transported organic and ions solutes which are converted into sugar inside the fruit [40], which is where foliar spraying the seaweed extract increases sugar biosynthesis and contributes to a higher TSS content [41]. The application of Se led to an increase in TSS (20%) in comparison with the control treatment. Similar results were also obtained in a case where Se was added as a nutritional solution [42]. It was reported that the use of Se led to enhancements of starch accumulation and positively affected soluble solid accumulation in ripe fruits [43]. Our results suggested that the application of seaweed extracts and Se may positively enhance TSS, thereby positively affecting consumer preferences. In the same line, Mohammadi Ghehsareh et al. [44] showed that the TSS content of tomatoes cultivated in perlite or date palm substrates had no significant differences. These results showed that the date palm peat can be used as a substrate for hydroponic systems with similar effects to coco peat substrate. Also, as it has suitable physical properties, low cost, and high availability [45], therefore, this substrate can be a good alternative to coco peat in hydroponic systems.

### 4.3. TTA, pH, and flavour index

The TTA is one of the most important quality attributes, which determines the storage life, the stability of ascorbic acid, and the flavour of tomato products [46]. Our study revealed that the application of different substrates had no significant effect on TTA and pH (Table 3). These results were similar to previous studies by Mohammadi Ghehsareh et al. [44] on tomatoes grown in date-palm and perlite substrates. We found that the usage of seaweed extract, Si, or Se, significantly increased TTA contents and pH in tomato fruits in comparison with the control treatment, whereas the highest TTA contents and pH belonged to plants treated with the seaweed extract (10%). This study confirmed earlier reports that the application of seaweed extract, Si, and Se led to enhancements in the TTA and pH in different plants such as strawberries [36] and mango [47]. It was demonstrated that the usage of seaweed extract stimulated the biosynthesis of organic acids in a differentiated way and decelerated the natural process of senescence of different fruits, since the decline in fruit acidity was almost associated with the utilization of acids in the respiratory process, due to the process of maturation and fruit ripening [47]. Consistent with the current results, Karagiannis et al. [48] showed the increase of titratable acidity after Si foliar application due to the influence of Si on the decrease of ethylene production and respiration. These results indicated that the high titratable acidity content in tomato fruits by the foliar application of seaweed extract, Si, or Se led to a decrease in the need for adding acidifiers to fruit juice [49].

The ratio of TSS to TA is ordinarily applied as a flavour index for tomato fruit. The flavour index is called the maturity index. Low amounts of flavour index show correlated with acid flavour while higher amounts of flavour index caused a mild flavour due to the excellent combination between acidity and sugar content. These results are coherent with Peripolli et al. [50] report. Mohammadi Ghehsareh et al. [44] showed that higher amounts of flavour index were obtained by using coco peat + perlite as substrate in comparison with palm peat + perlite, perlite, and palm peat substrates. The application of coco peat as substrate in hydroponic systems probably provided a suitable environment for root growth and development, while acting against drought stress and restricting water consumption, thereby improving the flavour index.

### 4.4. Vitamin C

Vitamin C, as an antioxidant, is one of the major nutritional quality indicators of fruits [51]. It was demonstrated that the amounts of vitamin C in plants cultivated on various substrates (i.e. coconut fiber, peat moss, perlite, and bark) varied considerably (14 to 32 mg/100g FW) [52]. Previous results confirmed further our results on the useful effect of palm peat substrate on the increase in vitamin C content in comparison with the effect of coco peat. Ahmad et al. [44] reported that the available potassium in palm peat was instantly higher than that in coco peat substrates. It was stated that an adequate amount of potassium is essentially required to improve ascorbic acid which, thus, increases the quality of tomato fruits [53].

### 4.5. Fruit volume and yield

Our results showed that the highest amount of fruit yield (FY) was observed in palm peat substrate in comparison with coco peat, but these results had no significant difference (Table 3). Similarly, Mohammadi Ghehsareh et al. [44] reported that the FY of greenhouse tomato was not affected by the usage of cocopeat, perlite, and palm peat substrates. The present study showed that fruit volume and FY of tomato plants were instantly influenced by foliar spraying with seaweed extract and Si. Various studies revealed that the usage of seaweed extract as a foliar spray or soil application increased the yield components in different crops [41, 54]. Seaweed extract included plant hormones (auxins, gibberellins, and cytokinins), trace elements (Zn, Cu, Fe, Mo, Ni, and Mn), amino acids, and vitamins [41]. It seems that the increase in fruit volume and FY by seaweed extract may be due to a higher increase in sink strength of the tomato fruits to attract more assimilates and water. Jarosz and Dzida [55] reported that the application of Si led to improvements in flowering and fruit set in tomatoes. In the present study, it seems that the enhancement of FY through foliar spraying with Si was explained by better photosynthesis, flowering, and fruit set [14]. Our results showed that the application of Se (4 and 10 mg L^-1^) significantly increased FY and FV in comparison with the control. The effects of Se on plants depended on the sensitivity of each species, the applied concentration, and the form of application [42]. It was reported that the application of Se in low dosages improved yield, while high doses of Se increased free radical production, causing oxidative stress [56]. It seems that the increase of resistance to oxidative stress can probably have key effects on the increase in tomato yield by foliar spraying with Se [57].

## 5. Conclusion

This research demonstrated that foliar application of seaweed extract, silicon, and selenium significantly enhanced the quality and quantity of tomatoes. In general, applications of seaweed extract (20%) and Si (75 mg/L) was more effective in improving total carbohydrate content, fruit firmness, TTA, pH, TSS, flavour index, and FY, compared with the rest of the treatments. In contrast to the foliar spray treatments, our results showed that the vitamin C content in fruits increased by the effect of date-palm peat substrate. The present study suggested that foliar spraying with seaweed extract and Si could enhance the quality and FY of tomatoes. Also, our results led to the recommendation that the application of palm peat can be a good alternative to coco peat in hydroponic systems.

## References

[1] Alves LR. Biochemical and structural alterations induced by selenium under cadmium stress in tomato plants. Universidade Estadual Paulista (UNESP). 2019.

[2] Farajpour M, Ebrahimi M, Amiri R, Nori SAS, Golzari R. Investigation of variations of the essential oil content and morphological values in yarrow (Achillea Santolina) from Iran. J Med Plants Res. 2011;5: 4393–4395.

[3] Sayed EG, Mahmoud AWM, El-Mogy MM, Ali MAA, Fahmy MAM, Tawfic GA. The Effective Role of Nano-Silicon Application in Improving the Productivity and Quality of Grafted Tomato Grown under Salinity Stress. Horticulturae. 2022;8: 293.

[4] Peris-Felipo FJ, Benavent-Gil Y, Hernández-Apaolaza L. Silicon beneficial effects on yield, fruit quality and shelf-life of strawberries grown in different culture substrates under different iron status. Plant Physiol Biochem. 2020;152: 23–31. doi: 10.1016/j.plaphy.2020.04.026 32361399

[5] Emad A-A, Yousry B, Elmahdy M, Mohamed R. Silicon supplements affect yield and fruit quality of cucumber (Cucumis sativus L.) grown in net houses. African J Agric Res. 2017;12: 2518–2523. doi: 10.5897/ajar2017.12484

[6] Zhu Z, Chen Y, Shi G, Zhang X. Selenium delays tomato fruit ripening by inhibiting ethylene biosynthesis and enhancing the antioxidant defense system. Food Chem. 2017;219: 179–184. doi: 10.1016/j.foodchem.2016.09.138 27765214

[7] Zhu Z, Zhang Y, Liu J, Chen Y, Zhang X. Exploring the effects of selenium treatment on the nutritional quality of tomato fruit. Food Chem. 2018;252: 9–15. doi: 10.1016/j.foodchem.2018.01.064 29478567

[8] Sabatino L, Ntatsi G, Iapichino G, D’Anna F, De Pasquale C. Effect of selenium enrichment and type of application on yield, functional quality and mineral composition of curly endive grown in a hydroponic system. Agronomy. 2019;9: 207.

[9] Saffan MM, Koriem MA, El-Henawy A, El-Mahdy S, El-Ramady H, Elbehiry F, et al. Sustainable Production of Tomato Plants (Solanum lycopersicum L.) under Low-Quality Irrigation Water as Affected by Bio-Nanofertilizers of Selenium and Copper. Sustainability. 2022;14: 3236.

[10] Xie Y, Su L, He Z, Zhang J, Tang Y. Selenium inhibits cadmium absorption and improves yield and quality of cherry tomato (Lycopersicon esculentum) under cadmium stress. J Soil Sci Plant Nutr. 2021;21: 1125–1133.

[11] Liu R, Deng Y, Zheng M, Liu Y, Wang Z, Yu S, et al. Nano selenium repairs the fruit growth and flavor quality of tomato under the stress of penthiopyrad. Plant Physiol Biochem. 2022. doi: 10.1016/j.plaphy.2022.05.026 35640519

[12] Salmi MS, Hesami M. Time of collection, cutting ages, auxin types and concentrations influence rooting Ficus religiosa L. stem cuttings. J Appl Environ Biol Sci. 2016;6: 124–132.

[13] Hesami M, Daneshvar MH, Lotfi A. In vitro shoot proliferation through cotyledonary node and shoot tip explants of Ficus religiosa L. Plant Tissue Cult Biotechnol. 2017;27: 85–88.

[14] Zhang Y, Yu SHI, Gong H, ZHAO H, LI H, HU Y, et al. Beneficial effects of silicon on photosynthesis of tomato seedlings under water stress. J Integr Agric. 2018;17: 2151–2159.

[15] Khan A, Kamran M, Imran M, Al-Harrasi A, Al-Rawahi A, Al-Amri I, et al. Silicon and salicylic acid confer high-pH stress tolerance in tomato seedlings. Sci Rep. 2019;9: 1–16. 31874969 10.1038/s41598-019-55651-4 PMC6930214

[16] Hernández-Herrera RM, Santacruz-Ruvalcaba F, Ruiz-López MA, Norrie J, Hernández-Carmona G. Effect of liquid seaweed extracts on growth of tomato seedlings (Solanum lycopersicum L.). J Appl Phycol. 2014;26: 619–628. doi: 10.1007/s10811-013-0078-4

[17] Almaroai YA, Eissa MA. Role of marine algae extracts in water stress resistance of onion under semiarid conditions. J Soil Sci Plant Nutr. 2020;20: 1092–1101.

[18] Hussain HI, Kasinadhuni N, Arioli T. The effect of seaweed extract on tomato plant growth, productivity and soil. J Appl Phycol. 2021;33: 1305–1314.

[19] Chen D, Zhou W, Yang J, Ao J, Huang Y, Shen D, et al. Effects of seaweed extracts on the growth, physiological activity, cane yield and sucrose content of sugarcane in China. Front Plant Sci. 2021;12: 659130. doi: 10.3389/fpls.2021.659130 34122479 PMC8189154

[20] Dhen N, ben Abed S, Zouba A, Haouala F, AlMohandes Dridi B. The challenge of using date branch waste as a peat substitute in container nursery production of lettuce (Lactuca sativa L.). Int J Recycl Org Waste Agric. 2018;7: 357–364. doi: 10.1007/s40093-018-0221-y

[21] Sardoei AS. Effect of different Media on growth, Sucker and Chlorophyll of Padanus spp in Under System Mist. Eur J Exp Biol. 2014;4: 285–288.

[22] Mohammadi Ghehsareh A. Effect of date palm wastes and rice hull mixed with soil on growth and yield of cucumber in greenhouse culture. Int J Recycl Org Waste Agric. 2013;2: 1–5. doi: 10.1186/2251-7715-2-17

[23] Hoagland DR, Arnon DI. The water-culture method for growing plants without soil: University of California. Circ Calif Agric Exp Stn. 1938;347: 32 pp.

[24] Valencia RT, Acosta LS, Hernández MF, Rangel PP, Robles MÁG, Cruz R del CA, et al. Effect of seaweed aqueous extracts and compost on vegetative growth, yield, and nutraceutical quality of cucumber (Cucumis sativus L.) fruit. Agronomy. 2018;8: 264. doi: 10.3390/agronomy8110264

[25] Kiely DE. Carbohydrate chemistry. Journal of Carbohydrate Chemistry. 1992. p. a–b. doi: 10.1080/07328309208016146

[26] Khan, Ahmad N, Malik AU, Saleem BA, Rajwana IA. Pheno-physiological revelation of grapes germplasm grown in Faisalabad, Pakistan. Int J Agric Biol. 2011;13: 791–795.

[27] Kader A, Stevens MA. Composition and Flavor Quality of Fresh Market Tomatoes as Influenced by Some Postharvest Handling Procedures. Journal of the American Society for Horticultural Science. 1978. pp. 6–13.

[28] Contreras-Calderón J, Calderón-Jaimes L, Guerra-Hernández E, García-Villanova B. Antioxidant capacity, phenolic content and vitamin C in pulp, peel and seed from 24 exotic fruits from Colombia. Food Res Int. 2011;44: 2047–2053. doi: 10.1016/j.foodres.2010.11.003

[29] Hong K, Xie J, Zhang L, Sun D, Gong D. Effects of chitosan coating on postharvest life and quality of guava (Psidium guajava L.) fruit during cold storage. Sci Hortic (Amsterdam). 2012;144: 172–178. doi: 10.1016/j.scienta.2012.07.002

[30] Huang Y, Lu R, Chen K. Prediction of firmness parameters of tomatoes by portable visible and near-infrared spectroscopy. J Food Eng. 2018;222: 185–198.

[31] Tarabih ME, EL-Eryan EE, EL-Metwall MA. Physiological and Pathological Impacts of Potassium Silicate on Storability of Anna Apple Fruits. Am J Plant Physiol. 2014;9: 52–67. doi: 10.3923/ajpp.2014.52.67

[32] Dehghanipoodeh S, Ghobadi C, Baninasab B, Gheysari M, Bidabadi SS. Effects of potassium silicate and nanosilica on quantitative and qualitative characteristics of a commercial strawberry (fragaria × ananassa cv. ‘camarosa’). J Plant Nutr. 2016;39: 502–507. doi: 10.1080/01904167.2015.1086789

[33] Malakouti MJ, Razaie H. The role of sulfur, calcium and magnesium on the quantity and improve the quality of agricultural products. Agric Educ. 2001.

[34] Hernández-Hernández H, Quiterio-Gutiérrez T, Cadenas-Pliego G, Ortega-Ortiz H, Hernández-Fuentes AD, Cabrera de la Fuente M, et al. Impact of selenium and copper nanoparticles on yield, antioxidant system, and fruit quality of tomato plants. Plants. 2019;8: 355. doi: 10.3390/plants8100355 31546997 PMC6843222

[35] Kumari R, Kaur I, Bhatnagar AK. Effect of aqueous extract of Sargassum johnstonii Setchell & Gardner on growth, yield and quality of Lycopersicon esculentum Mill. J Appl Phycol. 2011;23: 623–633. doi: 10.1007/s10811-011-9651-x

[36] Mousa S, Youssef S, Abdel A, Hamed F, Metwally A. Influence of Foliar Spraying of Seaweed Extract on Growth, Yield and Quality of Strawberry Plants. J Appl Sci Res. 2016;10: 88–94.

[37] Goyal P, Thind SK. Morphological parameters and carbohydrate accumulation of rice cultivars as influenced by seaweed extract application under aerobic conditions. An Int J Rice. 2015; 131.

[38] Rasouli F, Amini T, Asadi M, Hassanpouraghdam MB, Aazami MA, Ercisli S, et al. Growth and Antioxidant Responses of Lettuce (Lactuca sativa L.) to Arbuscular Mycorrhiza Inoculation and Seaweed Extract Foliar Application. Agronomy. 2022;12: 401.

[39] Pakrah M, Poorhashemi A, Mohamadzadeh P. Investigation and determination of the best strategy for sustainable development of nomadic areas of Iran based on the rights of local societies. Eurasia J Bi. 2020;14: 955–966.

[40] Nguyen-Quoc B, Foyer CH. A role for “futile cycles” involving invertase and sucrose synthase in sucrose metabolism of tomato fruit. Journal of Experimental Botany. Oxford University Press; 2001. pp. 881–889. doi: 10.1093/jexbot/52.358.881 11432905

[41] Abbas M, Anwar J, Zafar-Ul-Hye M, Khan RI, Saleem M, Rahi AA, et al. Effect of seaweed extract on productivity and quality attributes of four onion cultivars. Horticulturae. 2020;6: 28. doi: 10.3390/horticulturae6020028

[42] Puccinelli M, Malorgio F, Pezzarossa B. Selenium enrichment of horticultural crops. Molecules. 2017;22: 933. doi: 10.3390/molecules22060933 28587216 PMC6152644

[43] Aly RAM, Abdel-Halim KY. Effect of bio-fertilizer and foliar spray of selenium of growth, yield and quality of potato plants. Acad J Life Sci. 2020;6: 1–7.

[44] Ghehsareh AM, Borji H, Jafarpour M. Effect of some culture substrates (date-palm peat, cocopeat and perlite) on some growing indices and nutrient elements uptake in greenhouse tomato. African J Microbiol Res. 2011;5: 1437–1442.

[45] Borji H, Mohammadighesareh A, Jafarpour M. Effect of date-palm and perlite substrates on nutrients content and quality of tomato grown in soilless culture. Res Crop. 2012;13: 258–261.

[46] Bilalis D, Krokida M, Roussis I, Papastylianou P, Travlos I, Cheimona N, et al. Effects of organic and inorganic fertilization on yield and quality of processing tomato (Lycopersicon esculentum Mill.). Folia Hortic. 2018;30: 321–332. doi: 10.2478/fhort-2018-0027

[47] de Melo TA, Serra IMR de S, Sousa AA, Sousa TYO, Pascholati SF. Effect of ascophyllum nodosum seaweed extract on post-harvest ‘tommy atkins’ mangoes. Rev Bras Frutic. 2018;40. doi: 10.1590/0100-29452018621

[48] Karagiannis E, Michailidis M, Skodra C, Molassiotis A, Tanou G. Silicon influenced ripening metabolism and improved fruit quality traits in apples. Plant Physiol Biochem. 2021;166: 270–277. doi: 10.1016/j.plaphy.2021.05.037 34130037

[49] Cavichioli JC, Ruggiero C, Volpe CA. Caracterização físico-química de frutos de maracujazeiro-amarelo submetidos à iluminação artificial, irrigação e sombreamento. Rev Bras Frutic. 2008;30: 649–656. doi: 10.1590/S0100-29452008000300015

[50] Peripolli M, Da Silva ACF, Dornelles SHB, Sanchotene DM, Trivisiol VS. Use of seed+® and crop+® bioestimulants on the quality of tomato fruits under water stress. Rev Caatinga. 2020;33: 266–273. doi: 10.1590/1983-21252020v33n129rc

[51] Santos PHS, Silva MA. Retention of vitamin C in drying processes of fruits and vegetables—A review. Drying Technology. Taylor & Francis; 2008. pp. 1421–1437. doi: 10.1080/07373930802458911

[52] Sikorska-Zimny K, Kowalczyk W, Konopacki P, Hołownicki R. The dependent chemical composition of tomatoes grown in high plastic tunnels from the plant growth medium and the supply of heat. iaras.org. International Association of Research and Science; 2020. Available: http://www.iaras.org/iaras/journals/ijes.

[53] Kaur H, Bedi S, Sethi VP, Dhatt AS. Effects of substrate hydroponic systems and different N and K ratios on yield and quality of tomato fruit. J Plant Nutr. 2018;41: 1547–1554. doi: 10.1080/01904167.2018.1459689

[54] Tripathi P, Na CI, Kim Y. Effect of silicon fertilizer treatment on nodule formation and yield in soybean (Glycine max L.). Eur J Agron. 2021;122: 126172. doi: 10.1016/j.eja.2020.126172

[55] Jarosz Z, Dzida K. Effect of substratum and nutrient solution upon yielding and chemical composition of leaves and fruits of glasshouse tomato grown in prolonged cycle. Acta Sci Pol Hortorum Cultus. 2011;10: 247–258.

[56] Zięba P, Kała K, Włodarczyk A, Szewczyk A, Kunicki E, Sękara A, et al. Selenium and zinc biofortification of Pleurotus eryngii mycelium and fruiting bodies as a tool for controlling their biological activity. Molecules. 2020;25: 889. doi: 10.3390/molecules25040889 32079328 PMC7070737

[57] Babalar M, Mohebbi S, Zamani Z, Askari MA. Effect of foliar application with sodium selenate on selenium biofortification and fruit quality maintenance of ‘Starking Delicious’ apple during storage. J Sci Food Agric. 2019;99: 5149–5156. doi: 10.1002/jsfa.9761 31032929

